# Evidence-based bioethics: delineating the connections between science, practice, and values in medicine

**DOI:** 10.3325/cmj.2016.57.307

**Published:** 2016-08

**Authors:** Stjepan Orešković

**Affiliations:** Andrija Štampar School of Public Health, University of Zagreb School of Medicine, Zagreb, Croatia

## Diverse origins of bioethics

The origins of bioethics, self-defined as “science of survival”, are diverse (1). They can be traced back to the Code of Hammurabi (1754 B.C.), which introduced specific rules and drastic penalties for physicians in the cases of therapeutic failure. Using a connection between responsibility for a medical intervention (rules 215-225) and measurable outcome, the Code represents an early attempt to establish strict behavioral guidelines: “If a physician make a large incision with the operating knife, and kill him, or open a tumor with the operating knife, and cut out the eye, his hands shall be cut off” (2). There is a significant difference between the evidence collected to make a value judgment for a particular case and the evidence that serves in the process of testing a certain hypothesis about the nature of things (3). The Judgment of Solomon represents the model and the case for a peculiar bioethical method and approach in a “life-or-death situation” decision-making. When King Solomon of Israel was called to make a judgment regarding two women who both claimed to be the mother of a child, he employed a wise and intuitive method. He was tricking the “mothers” into revealing their true feelings. From a bioethical point of view, his task was to distinguish the right outcome from the wrong outcome without any empirical evidence. The episode has become an example of a middle ground argument (*argumentum ad temperantiam*), where an impartial judge displays wisdom in making a decision. Solomon was collecting evidence with a non-standard, non-epidemiological method for informed decision making. The Case of Re A (Separation of Conjoined Twins and a decision of the Court of Appeal of England and Wales) likewise demonstrates the complexity of bioethical decision-making (4). The Hippocratic Oath (500 B.C.E.) marks the beginning of Western ethical reasoning and decision-making in medicine. However, the well-known phrase “*primum non nocere*” (first, do no harm), which became the binding ethical rule of the utmost importance is not in the Hippocratic Oath. It comes from The History of Epidemics, which is part of the Hippocratic corpus (5). The same applies to the well-known principles of non-maleficence and beneficence “*salus aegroti suprema lex*” (well-being of the patient is the most important law).

From Hammurabi's Code to the moment when the German theologian Fritz Jahr published articles using the German term “Bio-Ethik” in 1927 (6) there were 3681 years of non-interrupted efforts directed toward establishing the ground for decision-making that would be ethical, objective, and life-saving. Finally, an important academic and professional “boost” for bioethics came with van Ransselaer Potter's “Bioethics, the Science of Survival” (7) and Callahan’s “Bioethics as a Discipline” (8). What where the key drivers for increased professional, public and institutional interest in bioethics in the late 1960s and early 1970s?

## Joint interests and parallel history of evidence-based medicine and evidence-based bioethics

The first and most important stimulus for establishing evidence-based bioethics was fostered by a series of important events in research and clinical medicine: the Harvard Definition of Brain Death (9), the Roe v. Wade case (10), the Karen Ann Quinlan case (11), and the Baby Doe case (12). The second important stimulus came from the institutional background. The Joint Commission for Accreditation of Healthcare Organizations that accredited hospitals in the USA introduced clinical ethics consultation as a must-have method for the improvement of quality assurance through the newly established hospital ethics committees (13).

The first to use the term “evidence-based medicine” was David M. Eddy in his work on population-level policies. He was also the first to link clinical practice guidelines and insurance coverage of new technologies with the idea of evidence-based bioethics. Two associations, the American College of Physicians and the American Heart Association, followed the initiative immediately in 1987 by publishing evidence-based guidelines for cardiovascular disease prevention. Another important step toward establishing evidence based medicine was made in the United Kingdom by Richard Smith's editorial in the *British Medical Journal* introducing the notion of evidence-based policies (14). Finally, five years later, the Cochrane Collaboration gathered a network of experts aiming to produce systematic reviews and guidelines. A similar tendency in the development of practice guidelines applies to evidence-based bioethics. A major difference in the intensity and pace of development is the level of specificity and a ten-to-fifteen-year delay. However, there are two similarities. One is the evaluation of ethical practices in the terms of effectiveness when issuing clinical practice guidelines and public health and population-based policies. The other is the introduction of epidemiological methods into individual patient-level decision-making (15).

A parallel history of evidence-based bioethics started in 1979, when Tom Beauchamp and James Childress published Principles of Biomedical Ethics (16), connecting efforts to resolve ethical issues in clinical medicine with the development of defined and concrete ethical principles – defining it as principalism (17). In the Belmont Report, principles of respect for persons, beneficence, and justice were identified as guidelines for responsible research using human subjects (18). However, efforts to regulate physicians' behavior through codes of ethics as specific ethical guidelines started already in 1847 in Philadelphia, when the American Medical Association established uniform standards for professional education, training, and conduct. The Code was adapted from the ethical code of conduct published in 1794 by Thomas Percival (19). After the World War 2, numerous international organizations joined the practice of developing bioethical codes for specific bioethical problems: the World Medical Association (WMA) accepted The Declaration of Helsinki (20); the Council for International Organizations of Medical Sciences (CIOMS), in collaboration with the World Health Organization (WHO), issued International Ethical Guidelines for Biomedical Research Involving Human Subjects (21); the Council of Europe (CoE) issued the Oviedo Convention – the Convention on Human Rights and Biomedicine (ETS No 164) (22); and the European Council and the European Parliament issued Directive 2001/20/EC (23). At the clinical level, practical approaches to ethical problem solving developed, and new institutions specialized for operational research in bioethics were established. The National Institute for Health Care Excellence became a model institution for quality improvement in health care through the development of evidence-based guidance that increasingly considers bioethical aspects of clinical decision-making (24). The Nuffield Council on Bioethics systematically identifies ethical questions raised by recent advances in biological and medical research, publishing reports and guidance on specific bioethical topics such as biological and health data, mitochondrial DNA disorders, Zika ethical considerations, genome editing and public dialogue, dementia, and invasive cosmetic procedures, just to mention a few (25).

## A case example of development of utilitarian bioethics: from Bentham's Felicific Calculus to World Happiness Report

How do we define and measure good and bad ethical outcomes in medicine and health care? How to even measure the bioethics of happiness? Pragmatic ethics attempts to use philosophical methods to identify the morally correct course of action concerned with legal issues in the life sciences. Ethical pragmatists, such as John Dewey, thought that norms, principles, and moral criteria were likely to be improved as a result of philosophical inquiry. Henry Sidgwick introduced the idea of motive or intent in morality, and Peter Singer conceptualized the idea of preference into moral decision making.

The idea of human happiness is a good example of the utilitarian theory approach to bioethics. The “greatest happiness principle” or the principle of utility, forms the cornerstone of Bentham's thought. Bentham tried to develop an operational concept for the scientific approach to human happiness by proposing a technical instrument “Felicific Calculus.” By “happiness,” he understood a predominance of “pleasure” over “pain.” In the Principles of Morals and Legislation he wrote: “The word *utility* does not so clearly point to the ideas of *pleasure* and *pain* as the words happiness, and *felicity* do: nor does it lead us to the consideration of the number, of the interests affected ” (26). Bentham’s disciple, John Stuart Mill, took a step further by trying to develop a system to measure pain and pleasure. Mill distinguished between higher and lower pleasures, understanding that certain human goods are irreducible to the calculation of the amount of pleasure or pain. Jeremy Bentham, or at least his auto-icon now on public display at the University College London, would be delighted to know that less than two centuries after his death, the United Nations Sustainable Development Solutions Network published the World 2015 Happiness Report. The report outlined the state of world happiness, causes of happiness and misery, and policy implications highlighted by case studies (27). The Gallup World Poll database was used as a rich source of information. Each variable from the Galupp Poll represents a population-weighted average score on a 0-t0-10 scale, and it is tracked over time and compared across more than 150 countries. These variables are healthy life expectancy, GDP per capita expressed in parity purchasing power (PPP), the freedom to make life choices, social support, generosity, and perceptions of corruption. Each country is compared to a hypothetical nation called Dystopia, a nation with the lowest averages for key variables and, along with the residual error, used as a regression benchmark (28). Psychologists, sociologists, economists, and statisticians analyze the feeling of happiness as related to general mental illness, the benefits of happiness, the relevance of bioethics, and policy implications, and link it to the Human Development Report (29).

## A critique of evidence-based bioethics

Empirical research in bioethics started at the end of the 20th century and was mostly influenced by developments in biological and clinical research, using methods from epidemiology and medical statistics. Writing during the first decade of the HIV/AIDS epidemic, Benjamin Freedman noted that “perception, rather than reality, controls the generation and resolution of ethical issues” (30). Freedman was referring to the debate on doctors' duties to provide care to AIDS patients and how they perceived the risk that patients might transmit the virus to them as vastly different from the actual risk involved. Halpern tried to establish an argument in support of the development and implementation of evidence-based bioethics by asking a logical question “but what ought to guide ethical deliberations once evidence becomes available?” (31) Value conflicts may emerge not only in clinical care or epidemiology of infectious diseases. The group of researchers from the Hastings Center demonstrated how values conflicts regularly emerged in health care organizations, public health, the regulatory context, and among payers. Conflicting situations may be resolved through a transparent public dialog about evidence involving patients, as well as public engagement (32). Societies also need to develop strategies for managing values conflicts, as well as any other health care-related complex behavioral and social situations.

After two decades of development, there is a long list of studies in favor of evidence-based bioethics. However, the scope of scientific evidence behind bioethics should not be limited exclusively to medical outcomes. As Goldenberg claimed “The qualitative, ethnographic, and phenomenological methods typically undertaken in empirical ethics are ranked low on the evidence-based hierarchy of knowledge…” (33). It is a consequence of the widespread practice and dominating convictions throughout the history of medicine that the primary goal of medical treatment should be “efficacy.” Such a reductionist approach creates space for health care policies that are frequently driven by ideologies and hidden agendas, rather than evidence. Influenced by ideology, many countries are enacting arbitrary health care, reproductive health, or pharmaceutical policies, and the only real strategies that might oppose such arbitrary policies are evidence-based bioethics principles implemented in everyday practice. The limits imposed on current research in empirical ethics by evidence-based approaches deserve further attention. There is a strong need for a discussion regarding the kind of normative (34) background and evidence that forms bioethical theories and informs bioethics.
